# Supplementary material for: Identification and functional analysis of non-coding regulatory small RNA FenSr3 in Bacillus amyloliquefaciens LPB-18
Source: PeerJ. 2023 May 15;11:e15236. doi: 10.7717/peerj.15236 (PMC10194069; doi:10.7717/peerj.15236)
Supplement: Supplemental Information 4 [file peerj-11-15236-s004.zip › KO/CK-vs-T1_map/map04933.html]

KEGG PATHWAY: AGE-RAGE signaling pathway in diabetic complications - Reference pathway


|  |  |
| --- | --- |
| **AGE-RAGE signaling pathway in diabetic complications - Reference pathway** |  |

[
Pathway menu
| Organism menu
| Pathway entry
| Show description
| User data mapping
]

Advanced glycation end products (AGEs) are a complex group of compounds produced through the non-enzymatic glycation and oxidation of proteins, lipids and nucleic acids, primarily due to aging and under certain pathologic condition such as huperglycemia. Some of the best chemically characterized AGEs include N-epsilon-carboxy-methyl-lysine (CML), N-epsilon-carboxy-ethyl-lysine (CEL), and Imidazolone. The major receptor for AGEs, known as receptor for advanced glycation end products (RAGE or AGER), belongs to the immunoglobulin superfamily and has been described as a pattern recognition receptor. AGE/RAGE signaling elicits activation of multiple intracellular signal pathways involving NADPH oxidase, protein kinase C, and MAPKs, then resulting in NF-kappaB activity. NF-kappa B promotes the expression of pro-inflammatory cytokines such as IL-1, IL-6 and TNF-alpha and a variety of atherosclerosis-related genes, including VCAM-1, tissue factor, VEGF, and RAGE. In addition, JAK-STAT-mediated and PI3K-Akt-dependent pathways are induced via RAGE, which in turn participate in cell proliferation and apoptosis respectively. Hypoxia-mediated induction of Egr-1 was also shown to require the AGE-RAGE interaction. The results of these signal transductions have been reported to be the possible mechanism that initates diabetic complications.

|  |  |  |
| --- | --- | --- |
| Reference pathway | 184% 150% 122% 100% 82% 67% 55% | 图片下载 |
